# Current Management and Future Directions for Pulmonary Arterial Hypertension Associated with Congenital Heart Disease

**DOI:** 10.3390/jpm14010005

**Published:** 2023-12-20

**Authors:** Ahmed K. Mahmoud, Mohammed Tiseer Abbas, Moaz A. Kamel, Juan M. Farina, Milagros Pereyra, Isabel G. Scalia, Timothy Barry, Chieh-Ju Chao, Francois Marcotte, Chadi Ayoub, Robert L. Scott, David S. Majdalany, Reza Arsanjani

**Affiliations:** 1 Department of Cardiovascular Medicine, Mayo Clinic, Phoenix, AZ 85054, USA; mahmoud.ahmed3@mayo.edu (A.K.M.);; 2 Department of Cardiovascular Medicine, Mayo Clinic, Rochester, MN 55905, USA

**Keywords:** pulmonary hypertension, congenital heart disease, sildenafil, bosentan, macitentan, epoprostenol, selexipag, sotatercept, ongoing trials

## Abstract

Current management of patients with congenital heart disease has increased their survival into adulthood. This is accompanied by potential cardiac complications, including pulmonary hypertension associated with congenital heart disease (PAH-CHD). PAH-CHD constitutes a challenging subgroup of pulmonary hypertension and requires expert management to improve quality of life and prognosis. Novel agents have shown a significant improvement in morbidity and mortality in patients with pulmonary arterial hypertension. However, the long-term effects of these medications on PAH-CHD patients remain somewhat uncertain, necessitating treatment plans largely founded on the clinical experience of the healthcare providers. The aim of this review is to summarize the current evidence and future perspectives regarding treatment strategies for PAH-CHD to help better guide management of this complex disease.

## 1. Introduction

Pulmonary hypertension (PH) is a significantly debilitating disease which is regularly observed in clinical practice. It was traditionally defined as a mean pulmonary artery pressure (mPAP) ≥ 25 mm Hg with a pulmonary vascular resistance ≥ 3 Wood units (WU) calculated with Thermodilution Cardiac output. Recently, the European Society of Cardiology (ESC)/European Respiratory Society (ERS) guidelines redefined PH as mPAP ≥ 20 mm Hg, along with the previously revised cardiac index ≥ 2 WU to emphasize earlier diagnosis and treatment [1].

PH has been classified into five groups according to the World Health Organization (WHO). The pathophysiology of PH is divided into three main mechanisms: that due to pre-capillary pulmonary arterial disease (group 1, or pulmonary arterial hypertension-(PAH)); that due to post-capillary disease, including left-heart disease (group 2) with passively elevated pulmonary venous pressure; and that due to a combination of pre- and post-capillary disease where passive pulmonary venous pressure elevation is associated with reactive pre-capillary pulmonary vasoconstriction. Patients with congenital heart disease (CHD) include a diverse group of lesions that may fall in group 1 or in group 2 [2]. Examples of group 1 lesions include intra- or extracardiac shunts, while examples of group 2 lesions include congenital mitro-aortic valve disease or left-sided obstructive lesions. Group 1 and 2 congenital heart lesions can best be defined hemodynamically through cardiac catheterization, by measuring, amongst several factors, the systemic and pulmonary cardiac index and the transpulmonary gradient (TPG), which is the difference between the mean (or diastolic) pulmonary artery pressure and the mean (or diastolic) wedge pressure. Pre-capillary disease or PAH is defined by a mean TPG > 12 mmHg (or diastolic TPG > 7 mmHg). This differentiation has a major impact on the treatments proposed. This review will principally cover group 1 congenital heart lesions associated with PAH. The incidence and prevalence of PAH-CHD have been reported to be 2.2 and 15.6 per 1 million, respectively, 58% of whom have Eisenmenger syndrome (ES) [3]. In the UK, the prevalence of PAH-CHD has been reported to be 5–10% of adults with congenital heart defects and a German study focusing on patients with septal defects reported that PAH-CHD was seen in 6.1% of the study population [4,5].

**Figure 1:**
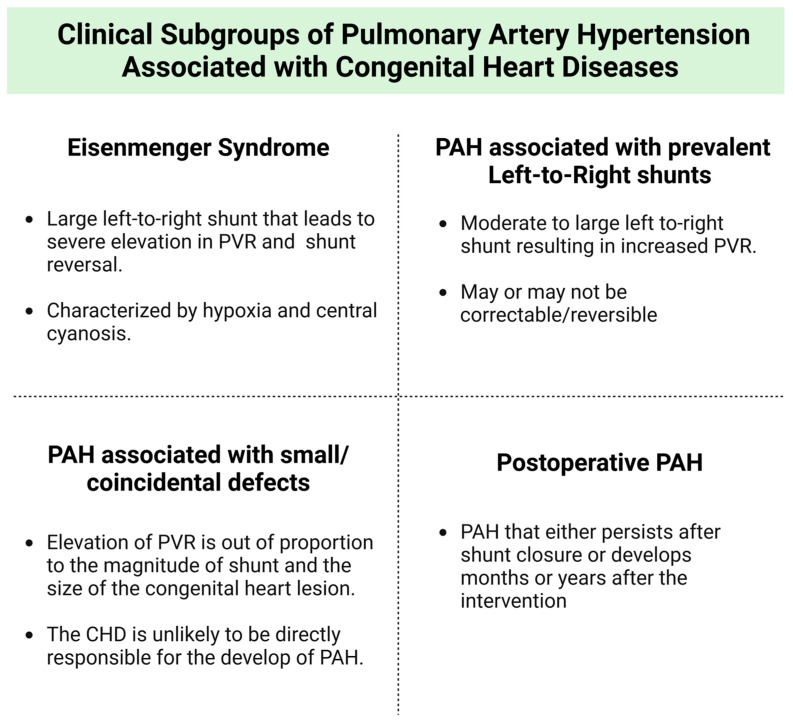
Clinical subgroup classification and pathophysiology, which help in better diagnosis and treatments plans. PAH: pulmonary arterial hypertension; PVR: pulmonary vascular resistance; CHD: congenital heart disease.

PAH-CHD has been classified into four clinical subgroups: ES, PAH-CHD associated with prevalent left-to-right shunts, PAH-CHD associated with small/coincidental defects, and postoperative PAH-CHD (Figure 1) [6]. This classification demonstrates underlying etiology and pathophysiology and helps recognize targeted treatment options. PAH-CHD constitutes a challenging subgroup of PAH and requires expert management to improve quality of life and prognosis. Novel agents have shown significant improvement in morbidity and mortality in patients with PAH. However, the specific effects of these medications on PAH-CHD patients remain uncertain and not clearly understood, mainly due to the scarce representation of this specific group of patients in clinical trials. The aim of this review is to summarize the current evidence and future perspectives regarding treatment strategies for PAH-CHD to help guide better management of this complex disease.

## 2. Pathophysiology

PAH-CHD constitutes a sequence of events that lead to the increase in pulmonary pressures, and it is usually the result of sustained intracardiac or extracardiac systemic-to-pulmonary shunts [7]. This leads to pressure and/or volume overload of the pulmonary circulation and ultimately leads to the remodeling of the pulmonary vascular bed due to shear stress and arterial endothelial damage. These vascular alterations further induce degeneration of the extracellular matrix and the release of growth factors (such as fibroblast growth factor), increase the production of vasoconstrictors such as endothelin-1, and decrease the production of vasodilators such as nitric oxide and prostacyclin [8]. Early in the process, these abnormalities include endothelial and medial hyperplasia, which can be reversible if the defects are fixed, but if the shunts are not properly treated, within the first 2 years of life, the remodeling will induce an irreversible increase in pulmonary vascular resistance (PVR) and mPAP. Once extensive pulmonary vessel thrombosis occurs and leads to the formation of plexiform lesions, it becomes no longer reversible. If mPAP exceeds systemic vascular pressure, the shunt can reverse and lead to hypoxemia and central cyanosis, as noted in ES [9].

ES is the most severe phenotype of PAH-CHD. It is the consequence of large systemic-to-pulmonary (left-to-right) shunts or bidirectional shunts which reverse to right-to-left shunting due to an exaggerated increase in PVR and pulmonary pressures. This significant increase in right-sided pressures very often triggers right ventricular hypertrophy. ES additionally leads to multisystem involvement, such as secondary erythrocytosis, thrombocytopenia, coagulation abnormalities, bleeding diathesis, thrombosis, propensity to infection, cerebrovascular events, renal dysfunction, right-heart failure, and early death [7]. Although this highlights the complexity and severity of this condition, patients with ES are considered to have a better prognosis than other forms of PAH [10,11].

## 3. Current Management

**Figure 2:**
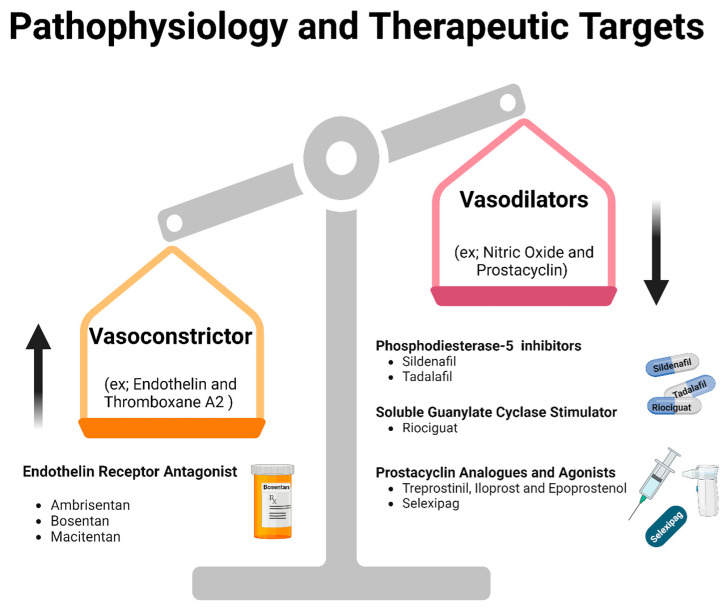
This diagram illustrates the imbalance between the vasodilator and vasoconstrictor mediators, which is the main cause of PAH. Furthermore, it shows the therapeutic lines targeted by each molecule.

As mentioned above, PAH-CHD occurs in part due to the imbalance between vasoconstrictor and vasodilatory factors in the pulmonary circulation (Figure 2). Medical treatments target these in three different pathways: the endothelin pathway, the nitric oxide (NO) pathway, and the prostacyclin pathway.

### 3.1. Eisenmenger Syndrome

#### 3.1.1. Endothelin-1 Pathway and ENDOTHELIN RECEPTOR ANTAGONISTS

Endothelin-1 (ET-1) is a very potent vasoconstrictor which is mainly released by endothelial cells. It works by binding to two different G-protein subtypes (ET-A, ET-B) in the pulmonary vessels’ smooth muscle cells and induces vasoconstriction and muscle cell proliferation [12].

Bosentan is an oral dual-endothelin-receptor (ET-A, ET-B) antagonist (ERA) which is the most studied ES targeted treatment [13]. The Bosentan Randomized Trial of Endothelin Antagonist THErapy-5 (BREATHE-5) was the first multicenter, double-blind, randomized, placebo-controlled trial studying the benefits of bosentan as a PAH-CHD targeted treatment. In total, 54 patients with ES were included in the study and randomized either to bosentan (37 patients) or placebo (17 patients) for 16 weeks. It demonstrated improved 6 min walk distance (6MWD) by 13% among patients in the bosentan group compared to the placebo group, which decreased by 3%. In addition, there were statistically significant reductions in PVR (9.3% reduction with bosentan vs. 5.4% increase with placebo) and mPAP (decreased by 5 mmHg with treatment vs. 0.5 mm Hg increase with placebo) and improved functional status [14].

An open-label continuation study followed BREATHE-5 and included 37 patients for 40 weeks of therapy; this investigation confirmed and maintained improvements in exercise and functional capacity with longer follow-up [15]. A major side effect included two patients (5.4%) with increases in liver aminotransferases to three times above the upper limit of normal. However, this side effect is dose-dependent and reversible after dose reduction or withdrawal. Accordingly, patients on bosentan should have their liver function monitored monthly [16].

The EARLY study (treatment of patients with mildly symptomatic pulmonary arterial hypertension with bosentan) was a double-blind, randomized, controlled trial which included 185 patients aged 12 years or older with WHO FC II PAH. Among the included patients, 32 of them had PAH-CHD. At 6 months follow-up, the mean PVR was reduced to 83.2% of the baseline value in the bosentan group and increased to 107.5% of the baseline value in the placebo group (*p* < 0.001) [17]. An open-label extension phase of this study was performed with a median bosentan treatment of 51 months. It showed that most patients exposed to long-term bosentan maintained or improved their functional class; however, 20% of cases discontinued bosentan treatment due to side effects, which were most commonly elevated liver enzymes, or failure of treatment and worsening of PAH [18].

A small retrospective study reviewed 18 adult patients with PAH-CHD (15 of them with ES) treated with bosentan. No significant increase in liver transaminases was seen, and patients showed both improvements in functional class and 6MWD compared to baseline [19].

Macitentan is the newest studied oral dual-ERA. The Macitentan in Eisenmenger Syndrome to Restore Exercise Capacity (MAESTRO) study included 226 patients with ES and functional class II–III who were randomized 1:1 to placebo or macitentan 10 mg once daily for 16 weeks. At baseline, 60% of patients were in WHO functional class II and 27% were receiving phosphodiesterase type-5 inhibitors (PDE-5is). The MAESTRO trial showed that there was no significant improvement in the primary endpoint of 6MWD in patients with ES receiving macitentan [20]. Additionally, no pertinent trends were observed for the secondary endpoints (improvement in WHO functional class). Among exploratory endpoints (NT-proBNP levels and changes in pulmonary vascular resistance index), this treatment decreased NT-proBNP levels and improved the PVR index.

Based on the current data, the AHA/ACC and ESC guidelines recommend bosentan as first-line medical therapy in patients with PAH-CHD who are not eligible for defect closure [1,21]. Of note, both of the newer ERA’s macitentan and ambrisentan are dosed once daily and neither require monthly testing of liver function tests, making them a more favorable alternative to bosentan.

#### 3.1.2. Phosphodiesterase-5 Inhibitors

PDE-5i therapy targets another pathway by blocking the PDE-5 enzyme which prevents cGMP and cAMP degradation and increases their levels in pulmonary smooth muscle cells, leading to relaxation and vasodilation of the pulmonary vascular bed [22].

Sildenafil and tadalafil are widely used in PAH patients. Their adverse reactions may include vomiting, headache, bronchitis, pyrexia, and diarrhea. In combination with nitrates, they can cause fatal hypotension [23]. In a systematic review of 36 studies with 2,999 participants (all with pulmonary hypertension followed for at least 14 weeks), 11 studies included patients with PAH-CHD. This study showed improvements in WHO functional class and 6MWD in the PDE-5i group versus placebo. Furthermore, the PDE-5i plus combination therapy showed additional improvement in 6MWD [24].

A preliminary observational study investigated 16 symptomatic ES patients for a daily dose of tadalafil during a 12-week follow-up. There was a significant reduction in PVR at 12 weeks (19.22 ± 8.23 to 17.02 ± 6.19 Wood units; *p* = 0.03). None of the patients had significant adverse effects from the treatment [25].

In a randomized study, 28 patients with ES were given tadalafil or placebo for 6 weeks followed by crossover to the other drug after a washout period of 2 weeks. All patients showed significant improvement in 6MWD (404.18 ± 69.54 m vs. 357.75 ± 73.25 m, *p* < 0.001), and tadalafil produced a significant decrease in PVR (−7.32 ± 1.58, *p* < 0.001) and WHO functional class (1.96 ± 0.18 vs. 2.14 ± 0.44, *p* = 0.025) [26].

#### 3.1.3. Soluble Guanylate Cyclase Stimulator

Soluble guanylate cyclase (sGC) stimulators are a class of drugs that works directly on the NO pathway; riociguat was the first drug approved in this class and works with dual mechanisms: (1) it enhances the binding between sGC and endogenous NO by stabilizing their bond, and (2) it directly stimulates sGC independently to NO (in contrast to PDE-5is, whose action is dependent on the endogenous NO) [27].

Switching to riociguat versus maintenance therapy with PDE-5is in patients with pulmonary arterial hypertension (REPLACE) was a multicenter, open-label, randomized, controlled trial that included 226 patients who were assigned to riociguat (n = 111) or to the PDE-5i (n = 115). In these groups, 24 and 19 patients had a diagnosis of PAH-CHD, respectively. The primary endpoint (defined as clinical improvement by week 24) was met by 45 (41%) patients in the riociguat group and 23 (20%) in the PDE5i group (OR 2.78, *p* < 0.001). The most frequently occurring adverse events in the riociguat group were hypotension (14%), headache (13%), and dyspepsia (9%) [28]. This study suggested that switching to riociguat from a PDE-5i could offer therapy escalation in patients with PAH.

Riociguat is dosed at 8 h intervals, making patient compliance more challenging. Also, care must be taken to make sure patients do not take riociguat with PDE-5is simultaneously, or profound hypotension may ensue.

#### 3.1.4. Prostacyclin Pathway

Prostacyclin is a potent antiplatelet and vasodilator molecule released from the endothelial cells of pulmonary vessels. It acts directly on the surface receptor known as “IP”, triggering the activation of adenylate cyclase enzyme that increases intracellular cAMP. This process results in the prevention of platelet aggregation, the relaxation of smooth muscles, the inhibition of smooth muscle cell proliferation, and the vasodilation of pulmonary vasculature [29,30]. The short half-life of prostacyclin makes its use in the treatment of PAH patients challenging [31].

Epoprostenol, iloprost, and treprostinil are synthetic prostacyclin analogues which can be administered by intravenous (I.V) or subcutaneous (S.C) routes. They could carry systemic side effects, and for the I.V. route, a permanent I.V. catheter needs to be placed, which may be associated with complications such as obstruction and infection [32]. Treprostinil and iloprost can be administered by inhalation, which maximizes the pulmonary therapeutic effects and minimizes the systemic effects [33,34,35].

A clinical trial investigated the role of I.V. epoprostenol in 20 patients with PAH-CHD who failed conventional therapy. The study showed that after one year of use of epoprostenol, mPAP decreased by 21% (*p* < 0.001), and there were improvements in the cardiac index (3.5 ± 2.0 to 5.9 ± 2.7 L/min/m^2^, *p* < 0.001) and PVR (25 ± 13 to 12 ± 7 WU, *p* < 0.001) [36].

An observational study interpreted the effect of S.C. treprostinil in adult patients with PAH-CHD. In terms of hemodynamics, PVR significantly decreased (18.4 ± 11.1 to 12.6 ± 7.9 WU congenital heart rate, *p* = 0.003). Clinical symptoms improved after 12 months, with increase in 6MWD by a mean of 114 m (*p* < 0.001) and a significant improvement in functional class [37].

In a 48-week clinical trial studying the effect of long-term iloprost nebulized treatment in patients with ES, a significant decrease in mPAP and PVR (all *p* < 0.05) was demonstrated. There was also a significant improvement in functional class (*p* = 0.006) and 6MWD (310.6 ± 44.7 to 399.7 ± 80.8 m, *p* < 0.001) [38].

Selexipag is a novel drug in the treatment of PAH which is a direct prostacyclin receptor agonist. A prospective cohort study included 34 adult patients with PAH-CHD, 21 of whom had ES and 11 had PAH after CHD correction. This study confirmed the clinical benefit of selexipag in patients with PAH after defect correction and showed that these patients had more clinical benefit than ES. Additionally, tolerability for selexipag was low in patients with ES [39].

Selexipag use in ES has also been described in case series. In a report of five cases with ES who started selexipag on a background of combination therapy, an improvement in functional class was described [40]. Another case series studied five patients, three of them with ES, revealing a significant improvement in WHO functional class, 6MWD, and an improvement in hemodynamics [41].

#### 3.1.5. Combination Therapy

The AMBITION trial, a double-blind randomization study that included PAH patients (including PAH-CHD), showed that initial combination therapy with ambrisentan and tadalafil resulted in a significantly lower risk of clinical failure events than monotherapy. Additionally, the OPTIMA trial (a prospective multicenter study enrolling patients with PAH, including PAH-CHD) demonstrated that initial double combination therapy with macitentan and tadalafil showed a significant improvement in cardiopulmonary hemodynamics, functional class, NT-proBNP, and risk profile in newly diagnosed patients, from baseline to week 16. These findings support the early prescription of double oral combination therapy with an ERA and PDE-5i to optimally treat PAH [42].

In a retrospective cohort study including 60 patients with PAH-CHD (32 with ES, 9 with coincidental shunts, 18 with postoperative PAH, and 1 with a significant left-to-right shunt), prostanoids were added to dual combination therapy at maximum doses. Triple therapy demonstrated a significant improvement in 6MWD, WHO functional class, and NT-proBNP levels at 2 years. The addition of prostanoids was especially favorable for patients with pre-tricuspid defects and non-ES [43].

### 3.2. PAH Associated with Prevalent Left-to-Right Shunts

Patients with unrepaired, moderate to large, systemic-to-pulmonary shunts and mildly to moderately elevated PVR constitute the second group of PAH-CHD. These patients are in an earlier stage pathophysiologically compared ES but require more complex management strategies due to a lack of evidence and their limited inclusion in clinical trials [44,45]. The decision to close the shunt or to use medical therapy depends on several factors, including the severity of PAH, increased PVR, and the location and size of the defect [46].

Patients with large post-tricuspid shunts (ventricular septal defect [VSD], patent ductus arteriosus [PDA]) usually develop pulmonary vascular disease in early childhood, which makes shunt closure inadvisable [45]. Patients with PAH and pre-tricuspid shunts (atrial septal defect [ASD]), however, are more challenging to manage because of the controversy in guidelines over shunt closure versus PAH-specific therapy [45,47]. This group usually requires additional testing to determine their operability [7,48]. Noninvasive testing such as with echocardiography may be sufficient for some patients, but the majority would benefit from right heart catheterization, which can assess pressures and estimate PVR using the Fick principle [1,7]. The most accurate estimation of PVR requires direct Fick with the measurement of oxygen consumption, but this is not available in most laboratories. Therefore, estimated oxygen consumption is used to calculate cardiac output with the indirect Fick method, which is a major source of bias [1]. This method has been shown to have low accuracy and is no longer recommended by the guidelines [49].

Although there is agreement between guidelines that shunt closure is contraindicated in a subset of patients with severe pulmonary vascular disease, the operability cut-off values for shunt closure vary and are not definitively established [50]. The AHA/ACC guidelines are less conservative in recommending closure, classifying the operability of the second group of PAH-CHD patients into three subgroups based on their baseline hemodynamics and response to acute vasodilator testing [3]. Shunt closure is advised for patients with PVR < 4 WU*m^2^, Rp/Rs < 0.3 and discouraged for patients with PVR > 8 WU*m^2^, Rp/Rs > 0.5 as they approach the Eisenmenger physiology [3,7]. The third group with PVR 4–8 WU*m^2^, Rp/Rs 0.3–0.5 is a “grey” zone that requires a case-by-case approach to weigh the risks and benefits of shunt closure versus targeted PAH therapy with periodic re-evaluation for operability [3,46]. The decision to operate needs expertise that considers the individual clinical characteristics of the patient (age, shunt location, comorbidities) as well as additional parameters such as the degree of reversibility and biventricular systolic and diastolic functions [48]. If more information is needed, a decrease in CO and/or an increase in RV (right ventricle) filling pressures on the balloon occlusion test would indicate against defect closure [46].

The ESC guidelines use more restrictive criteria for shunt closure with lower PVR thresholds [50]. The ESC also contraindicates closure for patients with exertional hypoxemia and recommends fenestrated closure only for ASD patients with moderate to severe PAH who respond to targeted therapy, while the AHA/ACC does not explicitly mention these options [1,50]. It is very important to identify patients who will normalize their hemodynamics after shunt closure as the persistence of PAH after shunt closure carries a poorer prognosis compared to other groups of PAH-CHD [47,51].

In inoperable patients, the optimal risk–benefit ratio of different treatment strategies depends on the timing and purpose of vasodilator therapy. An expectant strategy waits for the shunt to clearly reverse to right to left before using pulmonary vasodilators in an attempt to delay or avoid the long-term risk of heart failure and death associated with these drugs [52,53]. “Treat and repair”, “intent to repair”, and “treat to close” are different terms that describe similar strategies that include lowering the PVR first with pulmonary vasodilator therapy as a bridge to close the shunt, either by surgery or a device. Although these strategies may offer a cure for some patients, they are risky and require careful selection of patients and close monitoring of PVR. In addition, they lack the support of long-term data, and more research is needed to investigate their outcomes and risks [7,45,54,55].

### 3.3. PAH Associated with Small/Coincidental Defects

This group consists of patients with a small/coincidental defect (usually VSD < 1 cm or ASD < 2 cm) that is not considered to be the cause of PAH. They have a similar profile to idiopathic PAH, and closure of the defect is contraindicated as in ES [7]. The main treatment strategy is proactive disease targeting therapy (DTT). Lung or heart–lung transplantation may be considered if symptoms do not improve with optimal oral and intravenous therapy [56]. The EARLY study of bosentan by Galie et al. included a small number of patients with small/coincidental defects and there was a significant reduction in PVR in the overall population [17,57]. However, further studies with larger sample size are needed to better assess the efficacy of these drugs in this specific subgroup.

### 3.4. Postoperative PAH

This group comprises patients who develop or experience persistence or recurrence of PAH after defect closure. The clinical phenotype is often aggressive and has a poor prognosis, so prevention of this scenario is crucial [47,58]. Late diagnosis and closure are the most likely causes of this, but genetic predisposition may also play a role, especially when PAH occurs many years after defect correction [7]. Like the previous group, these patients resemble idiopathic PAH patients and need close monitoring and proactive DTT, with transplantation as a last resort [59]. For these two groups, upfront combination therapy (ERA and PDE-5i) is recommended for low/intermediate risk patients, and triple therapy (ERA, PDE-5i, and prostanoid) for high-risk patients [1,7]. Anticoagulants may also be indicated due to the prothrombotic nature of idiopathic PAH, which they resemble mechanistically [8].

There is some evidence regarding medical treatment for this subgroup of patients. The randomized, controlled GRIPHON study included 1156 patients with PAH to receive placebo or selexipag in individualized doses. Among the included cohort, 110 patients with corrected PAH-CHD were enrolled. Morbidity events included disease progression or worsening of PAH that resulted in hospitalization, initiation of parenteral prostanoid therapy or long-term oxygen therapy, or need for lung transplantation or balloon atrial septostomy. The results showed that selexipag may delay disease progression and be well tolerated in patients with PAH, including corrected PAH-CHD [60].

The Pulmonary Arterial hyperTENsion sGC-stimulator Trial-1 (PATENT-1) was a phase 3, double-blind study which included 443 patients with symptomatic PAH to receive riociguat or placebo for 12 weeks. It included 35 patients with postoperative PAH-CHD. There was significant improvement in 6MWD in the riociguat group. In addition, there were significant improvements in PVR, NT-proBNP levels, WHO functional class, time to clinical worsening, and Borg dyspnea score. The most common serious adverse event was syncope [61].

## 4. Future Directions and Ongoing Trials

At present, there are medical treatments for PAH-CHD that focus on decreasing vasoconstriction and inflammation in pulmonary vessels after remodeling of pulmonary vessels has already occurred. Recently, many preclinical and clinical trials have studied treatments that interfere with the molecular pathways involved in remodeling to prevent progression of the disease and reverse abnormal remodeling (Table 1). Some of these studies also investigated the capacity of specific agents to improve RV function.

Selonsertib is a small-molecule ASK1 (apoptosis signal-regulating kinase 1) or MAP3K5 (mitogen-activated protein 3 kinase 5) inhibitor that has effects on the abnormal remodeling driven by hyperplasia, hypertrophy, fibrosis, and extracellular matrix deposition in the pulmonary circulation leading to increase in PVR and subsequently pulmonary pressure. This drug is being studied in a multicenter, randomized, double-blind, placebo-controlled, phase II trial to determine the efficacy, safety, and tolerability in patients with PAH from different etiologies, including patients with repaired PAH-CHD who were on stable therapy (The ARROW trial). Preliminary results showed that using selonsertib in three different doses for 24 weeks in PAH patients who already were on the standard treatment did not lead to any significant results in primary or secondary outcomes, but the drug had no significant side effects or safety concerns [62].

Imatinib is a tyrosine kinase inhibitor that inhibits BCR-ABL tyrosine kinase [63,64]. In the IMPRES trial, a multicenter, double blind, placebo-controlled, phase III study, this agent has shown an improvement in 6MWD and PVR at 400 mg doses in PAH, including repaired PAH-CHD [64]. However, imatinib doses were not well tolerated and therefore current trials try to focus on achieving clinical benefits by using the lowest possible effective dose [63,64]. A phase I/II open-label, single-arm trial is trying to determine the best tolerated dose of imatinib in the range from 100 mg up to 400 mg in PAH patients, including PAH-CHD after ≥1 year repair of congenital systemic to pulmonary shunt [63]. An ongoing phase 2b/3 clinical trial is studying the effects of inhaled imatinib on PVR and 6MWD in PAH, including repaired PAH-CHD [65].

Seralutinib is a novel selective tyrosine kinase inhibitor that targets the PDGFRa/b, CSF1R, and c-kit pathways, which play a key role in the pathological inflammation and remodeling involved in PAH [66,67]. Seralutinib has demonstrated efficacy and safety in preclinical models [68,69]. The TORREY trial (and its open-label extension) is a randomized, double-blind, placebo-controlled, phase II trial seeking to determine the safety and change from baseline PVR after 24 weeks of receiving inhaled seralutinib in PAH patients who are WHO FC II or III, including repaired PAH-CHD with simple systemic-to-pulmonary shunt [66,70]. Another phase 3, randomized, double-blind, placebo-controlled study is ongoing to determine the safety and change in 6MWD from baseline after 24 weeks of inhaled seralutinib in PAH patients, including repaired PAH-CHD [71].

Decreased activity of the AMP-activated protein kinase (AMPK) pathway has been linked with abnormal remodeling in PAH and metformin, which has significant activatory effects on the AMPK pathway, has shown promising results in vitro [72,73,74]. A prospective randomized study has suggested efficacy of adding metformin to bosentan for PAH-CHD patients with WHO FC II or III, excluding ES and those who developed PAH after defect correction. Bosentan–metformin combination therapy led to significant improvement in 6MWD and PVR index compared to bosentan alone [75].

Another aspect of treating PH is to reduce myocardial stress response from the high pulmonary pressures to prevent right heart dysfunction [76]. Recent studies have proposed the association of H2 antagonism with decrease in cardiomyocytes apoptosis, fibrosis, and improve in the RV function [77,78]. An ongoing phase 2, single-center, randomized placebo-controlled trial in adults with PAH including patients with PAH-CHD is evaluating the effects of famotidine in improving 6MWD, NYHA functional class, and decreasing BNP levels [76].

Multiple preclinical and animal models have associated low levels of dehydroepiandrosterone (DHEA) with severe PAH and mortality [79]. DHEA has been suggested to have cardioprotective effects on cardiomyocytes, and improve RV function [79,80]. A phase II, randomized, crossover, clinical trial is ongoing to determine the safety and effects of DHEA on RV longitudinal strain and RV ejection fraction measured by cardiac MRI compared to placebo in PAH patients, including PAH-CHD with congenital systemic-to-pulmonary shunt [81].

Recent data suggest an important role of serotonin in PAH pathogenesis, as serotonin synthesis is increased in the dysfunctional endothelial cells [82,83]. Rodatristat is a peripheral tryptophan hydroxylase 1 inhibitor that targets the serotonin synthesis pathway. ELEVATE2 is a phase 2, dose-ranging, randomized, double-blind, placebo-controlled, multicenter study of rodatristat ethyl in PAH patients including patients with repaired PAH-CHD. The purpose of this study is to evaluate the effects of adding this unique medication to the standard of care [82].

Pentoxifylline is a PDE-i which inhibits TNF and leukotriene synthesis, reduces inflammation, has hemorheological properties, and enhances the levels of thrombomodulin. This agent is being studied in a randomized interventional study as an adjunct therapy to routine treatment in patients with ES. The study seeks to evaluate effects on thrombomodulin levels and impact on physical capacity, SaO_2_, and right ventricular function [84].

**Figure 3:**
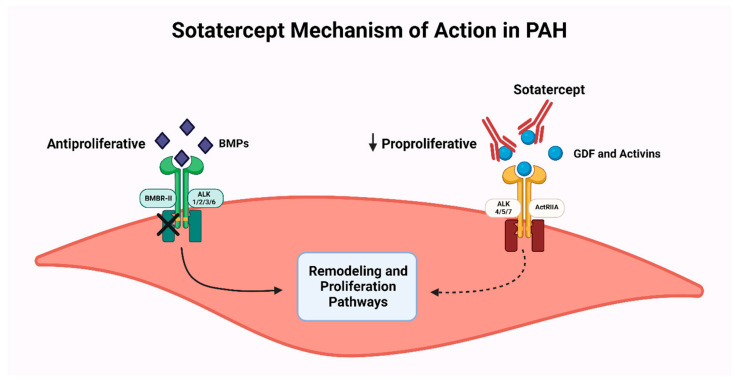
Sotatercept acts like a decoy receptor to ActRIIA ligands (activin and GDF) and prevents them from binding to their receptors. This restores the balance between antiproliferative and pro-proliferative signals favoring apoptotic and antiproliferative effects. ALK: anaplastic lymphoma kinase; BMP: bone morphogenic protein; BMPR2: bone morphogenic protein receptor type2; GDFs: growth differentiation factors; ActRIIA: activin receptor type-2A.

Mutations affecting the bone morphogenic protein type 2 (BMPR2) receptor have been associated strongly with hereditary PAH, and BMPR2 has also been linked with other subtypes of PAH, including PAH-CHD. BMPR2 plays a critical role in maintaining endothelial function and the balance between pro-proliferative agents (activin and growth differentiation factors (GDF)) and anti-proliferative agents (BMP2) [85,86,87]. Sotatercept is a decoy receptor to ActRIIA ligands (activin and GDF) and prevents them from binding to their receptors. This restores the balance between anti-proliferative and pro-proliferative signals favoring apoptotic and antiproliferative effects (Figure 3) [87]. A phase 2, double-blind, placebo-controlled, randomized study (PULSAR) has shown the efficacy of adding sotatercept in two different doses to the standard of care compared to placebo in patients with PAH, including patients with corrected congenital shunts. Another phase 3, randomized, double-blind, placebo-controlled, multicenter, parallel-group study (STELLAR) enrolled 323 subjects with symptomatic PAH, including 16 patients with corrected PAH-CHD. This study has shown efficacy in improving exercise capacity and NTproBNP levels. There was also a significant difference in the distribution of time to first occurrence of death or nonfatal clinical worsening event in the sotatercept and placebo groups (*p* < 0.001) [86]. An ongoing phase 3, randomized, double-blind, placebo-controlled study (ZENITH) is evaluating the effect of sotatercept plus the maximum standard therapy for 43 months on time to first confirmed morbidity or mortality events in PAH patients, including patients with corrected simple, congenital systemic-to-pulmonary shunts [88].

**Figure 4:**
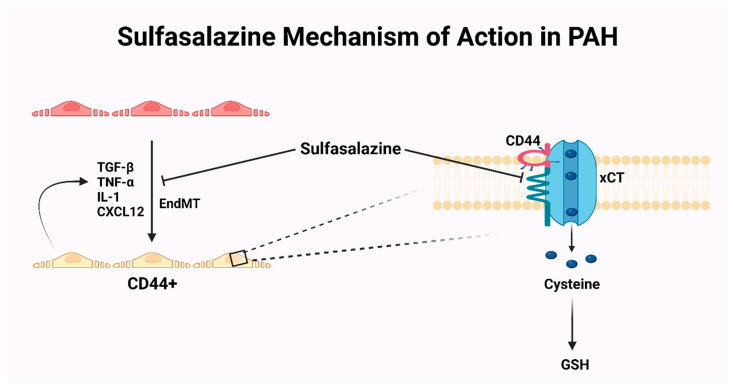
Inflammatory cytokines induce EndMT which in turn produces CD44+ mesenchymal cells. These cells, in turn, produce more cytokines and have the capability of proliferation and resistance to apoptosis because CD44 stabilizes xCT on cell membranes and enhances its function in transporting cysteine into the cell for GSH synthesis. PAH: pulmonary arterial hypertension; CD44: cluster of differentiation 44; GSH: reduced glutathione; TNF-a: tumor necrosis factor-alpha; TGF-b: transforming growth factor-beta; IL-1: interleukin1; CxCL12: C-X-C Motif Chemokine Ligand 12; EndMT: endothelial-to-mesenchymal transition; xCT: cysteine transporter.

A critical component of pulmonary remodeling is endothelial-to-mesenchymal transition (EndMT), which induces expression of specific CD44 isoforms (cd44v8-10) on the new cells [89,90,91]. This enhances cellular proliferation and promotes resistance to apoptosis. Sulfasalazine is a potential new agent for PAH that showed promising results in animal models in decreasing pulmonary vascular remodeling and CD44v+ cells given its anti-inflammatory effects and interference with CD44v–xCT axis (Figure 4) [89,90]. An ongoing phase II, randomized, parallel-group, placebo-controlled pilot study is evaluating the safety and effects of adding sulfasalazine to ambrisentan on 6MWD and time to first confirmed clinical adverse event in PAH patients, including those with PAH-CHD [92].

Apabetalone is a novel agent with a unique mechanism of action that inhibits BRD4 (bromodomain-containing protein 4), which is a histone deacetylase inhibitor (HDAC) upregulated in abnormal pulmonary cells [93,94]. An ongoing phase II, double-blind, parallel-group, placebo-controlled trial is evaluating the effects of apabetalone on PVR, 6MWD, NT-proBNP, and WHO functional class in 72 patients with PAH, including patients with simple CHD corrected for more than 1 year before inclusion [95].

Tacrolimus (FK-506) is an immunosuppressant agent that showed efficacy in preclinical studies in restoring normal BMPR2 signaling by binding to FK-binding protein-12 (FKBP12), a known repressor of BMPR2 signaling [96]. However, the TransformPAH study, which is a randomized, placebo-controlled, phase II trial, showed that low doses of tacrolimus failed to show positive effects on pulmonary parameters and exercise capacity in PAH patients including PAH-CHD [97].

mTOR is another pathway that has been linked to the abnormal proliferation and remodeling in PAH. Targeting this pathway with mTOR inhibitors, such as albumin-bound sirolimus (rapamycin), is being studied in phase I/Ib trials to determine its safety profile and toxicities in patients with PAH, including PAH-CHD (repaired greater than 1 year prior to screening) after being well studied to be safe and beneficial in animal models [98,99,100].

## 5. Conclusions

PAH-CHD is a debilitating disease with a challenging management due to its complex pathophysiology and the limited available evidence. Recent success in clinical trials provided evidence for PAH patients regarding novel agents and treatment strategies; however the majority of these trials underrepresented the PAH-CHD subgroup. Therefore, recommendations are usually extrapolated from general indications for overall PAH patients or are based on the experience of clinical providers. Ongoing and prospective studies are focusing on reversing the abnormal pulmonary vascular remodeling by targeting multiple potential pathways involved in PAH-CHD pathogenesis. The results of these trials will help better guide the management of this population.

## Figures and Tables

**Table 1:** A summary for the ongoing trials and mechanism of action of the drugs.

Medication	Mechanism of Action	Trial	Primary Endpoint
Selonsertib	ASK1(MAP3K5) inhibitor	(ARROW) NCT02234141	PVR
Imatinib	BCR-ABL tyrosine kinase inhibitor	(IMPAHCT) NCT05036135	PVR 6MWD
		(PIPAH) NCT04416750	MTD and PVR
Seralutinib	Selective tyrosine kinase inhibitor on PDGF Ra/b, CSF1R, and c-kit pathways	(PROSERA) NCT05934526	6MWD
		(TORREY) NCT04456998	PVR and adverse events
Pentoxifylline	PDE-i that inhibits TNF and leukotrienes synthesis, increases thrombomodulin. Hemorheological agent	NCT05611268	Thrombomodulin levels
Metformin	AMPK activator	Prospective randomized study	6MWD Endothelin-1 levels
H2 antagonists	Ameliorates RV strain	(REHAB-PH) NCT03554291	6MWD
DHEA	Protective effects on cardiomyocytes by reducing G6PD-derived NADPH	(EDIPHY) NCT03648385	RV longitudinal strain
Rodatristat	Peripheral tryptophan hydroxylase-1 inhibitor	(ELEVATE2) NCT04712669	PVR
Sotatercept	Decoy receptor for ActRIIA ligands to restore the balance between TGF-b superfamily members	(STELLAR) NCT04576988	6MWD and adverse events
		(PULSAR) NCT03496207 (ZENITH) NCT04896008	PVR and adverse events Morbidity and mortality
Sulfasalazine	Interference with CD44v–xCT axis	NCT04528056	Clinical adverse events
Spironolactone	Aldosterone antagonist, decreases remodeling	NCT01712620	6MWD
Apabetalone	Alters genetic transcription by inhibiting BRD4. Supportive effect on overloaded RV	(APPROACH-2) NCT04915300	PVR
Tacrolimus (FK-506)	Restores BMPR2 signaling	(TransForm PAH) NCT01647945	safety
albumin-bound sirolimus (Rapamycin)	mTOR inhibitor	NCT02587325	MTD and DLT

ASK1: apoptosis signal-regulating kinase 1; MAP3K5: mitogen-activated protein 3 kinase 5; PVR: pulmonary vascular resistance; BCR: breakpoint cluster region protein; ABL: Abelson murine leukemia viral oncogene homolog; 6MWD: 6 min walking distance; MTD: maximum tolerated dose; PDGFRa/B: platelet-derived growth factor receptor alpha and beta; CSF1R: colony-stimulating factor-1 receptor; C-Kit: receptor tyrosine kinase; PDE-i: phosphodiesterase inhibitor; TNF: tumor necrosis factor; AMPK: adenosine monophosphate-activated protein kinase; H2: histamine type 2 receptor; RV: right ventricle; DHEA: dehydroepiandrosterone; G6PD: glucose6phosphate dehydrogenase; NADPH: nicotinamide adenine dinucleotide phosphate hydrogen; ActRIIA: activin receptor type-2A; TGF-b: transforming growth factor-beta; CD-44v: cluster of differentiation44 variant; xCT: cysteine transporter; BRD4: bromodomain-containing protein4; FK-506: fujamycin-506; BMPR2: bone morphogenic protein receptor type2; mTOR: mammalian target of rapamycin; DLT: dose-limiting toxicity.

## Data Availability

No new data were created or analyzed in this study. Data sharing is not applicable to this article.
